# Adherence intervention for HIV-infected persons who use drugs: adaptation, open trial, and pilot randomized hybrid type 1 trial protocol

**DOI:** 10.1186/s13722-018-0113-5

**Published:** 2018-04-02

**Authors:** Kasey Claborn, Sara Becker, Don Operario, Steve Safren, Josiah D. Rich, Susan Ramsey

**Affiliations:** 1 Department of Psychiatry, The University of Texas at Austin Dell Medical School, 1912 Speedway Austin, TX USA; 2 Department of Medicine, Alpert Medical School of Brown University, Providence, RI USA; 3 Department of Psychiatry and Human Behavior, Alpert Medical School of Brown University, Box G-BH, Providence, RI 02912 USA; 4 Center for Alcohol and Addiction Studies, Brown University School of Public Health, 121 South Main Street, Box G-121-5, Providence, RI 02912 USA; 5 Department of Psychology, University of Miami, Coral Gables, FL USA; 6 The Center for Prisoner Health and Human Rights, The Miriam Hospital, Providence, RI 02906 USA

**Keywords:** HIV/AIDS, Substance use, Adherence, Motivational interview, Mixed methods, Protocol

## Abstract

**Background:**

HIV-infected people who use drugs (PWUD) exhibit the highest rates of non-adherence to antiretroviral therapy (ART) among people living with HIV. This contributes to poor treatment outcomes, increased morbidity and mortality, and HIV transmission. However, current interventions fail to address the unique barriers to adherence faced by this population. Life Steps is a brief, single session intervention that demonstrated increased ART adherence among HIV-infected individuals. This study protocol seeks to improve clinical practice by adapting Life Steps for HIV-infected PWUD and adding a brief motivational intervention addressing drug use. This intervention will incorporate educational, motivational, and behavioral skills components specifically aimed at improving adherence and linkage to substance use treatment among HIV-infected PWUD.

**Methods:**

This project will consist of three phases using a mixed-methods approach. *In Phase 1*, qualitative interviews with HIV-infected PWUD and community providers, coupled with feedback from an expert review panel, will be used to enhance the existing Life Steps manual and interventionist training protocol. *In Phase 2*, the prototype will be pilot tested and qualitative exit interviews with HPWUD will identify the strengths and limitations of the intervention. Data regarding feasibility, acceptability, and barriers to delivery will guide modifications to finalize a modified Life Steps-Drug Use (LS-DU) protocol. *In Phase 3*, a pilot type 1 hybrid effectiveness-implementation trial will examine the effectiveness of LS-DU relative to a health education intervention control condition on ART adherence and viral load data at 1-, 3-, and 6-months. Data regarding clinic readiness for implementation and intervention sustainability potential will be collected.

**Discussion:**

This protocol will adapt and evaluate an intervention to improve adherence among HIV-infected PWUD. Results of this study will provide significant data on the acceptability, initial effectiveness, and sustainability potential of an adherence intervention for a high risk and underserved population.

*Trial registration* NCT02907697

## Background

Illicit drug use remains a common and significant problem among people living with HIV (PLWH). Drug use is one of the most significant barriers to antiretroviral therapy (ART) adherence, contributing to suboptimal treatment outcomes and increased transmission of HIV. As suggested by the syndemics model [1], multiple morbidities such as drug use and HIV act synergistically to produce poorer health outcomes. Over 81% of persons living with HIV report a history of illicit drug use and nearly one in four meet diagnostic criteria for a severe substance use disorder [2]. Drug use remains prevalent among PLWH enrolled in primary care with one study showing that 24% report marijuana use, 9% report amphetamine use, 8.5% report crack cocaine use, 10% report polydrug use, and almost 3% report injection drug use in the preceding 3 months [3]. This study was conducted using the CFAR Network of Integrated Clinical Systems (CNICS) data which consisted of academically affiliated HIV clinics; thus, it is possible that these are underestimates of active illicit drug use. These data demonstrate that sustainable interventions and risk reduction programs addressing substance use are needed within the HIV clinical setting.

People who use drugs (PWUD) experience unique challenges with HIV treatment and adherence. HIV-infected PWUD are more likely to experience delayed treatment, suboptimal service utilization, increased rates of HIV transmission in the community, and inferior treatment outcomes [4–6]. Relative to PLWH who do not use drugs, HIV-infected PWUD are more than twice as likely to be non-adherent to ART [7]. Both interpersonal and social factors limit adherence in this population: HIV-infected PWUD are less likely to have primary care providers or strong patient-provider relationships and have lower levels of social support [7, 8]. Socioeconomic factors prevalent in PLWH, including lack of stable housing and medical insurance, poor education and low literacy, and low health literacy pose further barriers to adherence. HIV-infected PWUD are also more likely to have medical (e.g., hepatitis C, tuberculosis) and psychiatric (e.g., depression, anxiety) comorbidity, neurocognitive impairment, and increased risk for drug overdose, which contribute to reduced adherence and poorer health outcomes [8].

### Prior adherence interventions for HIV-infected people who use drugs

Several intensive treatment approaches have been shown to improve adherence among HIV-infected PWUD including the integration of HIV care and intensive drug use treatment, directly administered ART doses at the clinic, contingency management, and peer-driven interventions [6]. Although these interventions are effective in the short-term, adherence improvements are frequently not maintained after the direct observation has been terminated [9] and such intensive interventions may not be feasible or sustainable in clinics with limited resources, which dampens the potential for widespread implementation [10]. Additionally, these interventions do not teach HIV-infected PWUD the behavioral skills needed to maintain adherence once it is achieved. Behavioral interventions overcome these limitations by teaching adherence and problem-solving skills, using brief motivational interviewing (BMI) and cognitive behavioral approaches to sustain behavior change. These interventions have been successfully augmented with booster sessions and delivered by trained clinic nurses and staff. This approach has shown promise in promoting ART adherence and self-efficacy to adhere among PLWH, but evidence is severely limited by use of small sample pilot and case studies. To date, only one behavioral adherence intervention—the Life Steps intervention—has been evaluated in HIV-infected PWUD.

Life Steps was developed by Safren and colleagues using motivational interviewing and cognitive behavioral techniques to teach the following: facts about ART and HIV, problem-solving around transportation to clinic appointments and obtaining medications, skills to improve communication with healthcare providers, strategies to cope with side effects, development of a daily medication schedule, storage of medications away from home, reminder cues for pill-taking, and responding to slips in adherence. This model consists of a brief, single session intervention that has been evaluated as both a stand-alone intervention and an adjunct to more intensive cognitive behavioral therapy (CBT). Data from multiple studies indicates that Life Steps and similar interventions are associated with increased adherence relative to self-monitoring in PLWH [7], though meta-analyses suggest that these effects are modest across studies [11, 12].

### Limitations of life steps with HIV-infected people who use drugs

Prior research on the Life Steps protocol with HIV-infected PWUD is subject to several limitations. First, only one study to date has evaluated Life Steps among a drug using population and this study focused on depressed injection drug users who are unlikely to be representative of the full range of HIV-infected PWUD [13]. Additionally, both study conditions received Life Steps; the experimental condition also received CBT for Adherence and Depression which consisted of seven CBT-focused modules. Second, no prior studies have examined which specific components of the Information-Motivation-Behavioral Model are likely to be most relevant for HIV-infected PWUD and if additional skills modules related to illicit drug use should be incorporated. Third, the Life Steps protocol focuses on skills acquisition and behavior modification related to HIV medication taking behavior, but does not address substance use or linkage to addiction treatment. Brief motivational interventions (BMIs) are especially well suited to this population, as both adherence to HIV treatment and drug use have been related to patient motivation for change [14]. Finally, there has been virtually no research examining barriers and facilitators of the implementation of behavioral ART adherence interventions within real-world clinics. Such research is essential in order to accelerate the translation of research into practice and improve the outcomes of this hard to reach, high risk population.

### Protocol objective and specific aims

The overarching objective of the current protocol is to adapt a behavioral adherence intervention for HIV-infected PWUD and obtain data on its preliminary effectiveness and implementation potential. The proposed project consists of three phases (see Table 1). *In Phase 1*, qualitative interviews with HIV-infected PWUD and community providers, coupled with feedback from an expert review panel, will be used to adapt the existing Life Steps manual and community clinician training protocol. *In Phase 2*, qualitative data from phase 1 will inform development of the intervention manual. The protocol will be pilot tested and in-depth qualitative interviews with HIV-infected PWUD and community clinicians will be used to make final refinements to the adapted Life Steps-Drug Use (LS-DU) model. *In Phase 3*, a pilot type 1 hybrid effectiveness-implementation trial will examine the effectiveness of LS-DU relative to a health education control condition on ART adherence and viral load data at 1-, 3-, and 6-months. Data regarding clinic readiness for implementation and intervention sustainability potential will be also be collected. Combined, these three phases will address three study aims:

**Table 1:**
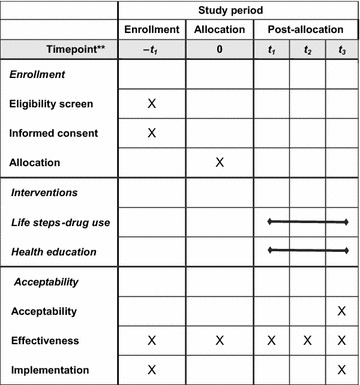
Clinical trial schedule of enrollment, interventions, and assessments

***Aim 1***: To adapt the Life Steps intervention by incorporating a BMI focused on linkage to substance use treatment, and tailoring the skills to meet the unique needs of HIV-infected PWUD. ***Aim 2***: To standardize the adapted intervention into a manual and train community-based intervention facilitators. ***Aim 3***: To obtain data on the preliminary effectiveness and implementation potential of the adapted intervention relative to a health education control.

## Methods

### Participants

A total of up to 84 patients and 35 clinicians will participate across three phases: 6 patients and 29 clinicians participated in Phase 1 (qualitative interviews), 18 patients and 6 clinicians will be recruited in Phase 2 (pilot testing), and 60 patients will be recruited in Phase 3 (pilot randomized hybrid trial). The primary objective for Phase 3 will be to determine a reasonable effect size for LS-DU rather than to determine statistical significance between groups. With 30 participants per group and effect size range of d = .40 to .60, power to detect the difference ranges from .54 to .75. With N = 60 we will have .80 power to detect effect sizes in the medium-large range above d = .65. Effect size estimates will include odds ratios for ART adherence and squared semi partial correlations for repeated continuous measures of putative mechanisms.

#### Inclusion criteria

Patients will be eligible for each phase of the study if they are (1) ≥ 18 years of age, (2) HIV-infected as confirmed by medical record review, (3) prescribed an ART regimen, and (4) meet DSM-V criteria for a substance use disorder (other than tobacco, marijuana, and alcohol) over the past 90 days. Although heavy alcohol consumption affects ART adherence and cannabis use has demonstrated mixed results in the literature, we chose to narrow the target population to illicit drug use to have a more homogenous sample for this pilot trial. In Phase 3 (pilot randomized trial), participants must also report < 100% adherence to ART regimen and have experienced a detectable viral load (> 20 copies/mL) within the last 6 months. Our eligibility criteria for a substance use disorder is broad given the LS-DU will focus on improving ART adherence and linking patients to appropriate substance use treatment resources. Our goal is to create an intervention that is easily implemented in clinical settings. We will examine qualitative data from Phase 1 to assess the potential need for the intervention to take a more narrow approach and adjust the manual development based on these data.

Clinicians were eligible to participate in Phase 1 provider interviews if they were: over the age of 18, employed at a local HIV clinic or substance use treatment facility, and had > 6 months experience working with patients living with HIV and/or patients with a substance use disorder.

#### Exclusion criteria

Exclusion criteria for all study phases include issues that jeopardize informed consent, including cognitive impairments, active psychosis, current suicidal ideation, and not being fluent in English. Patient participants will be administered a brief measure of capacity to consent [15].

### Study enrollment and randomization

In accordance with institutional review board procedures, participants will be recruited from an academic affiliated HIV treatment center which is funded by the Ryan White program and situated in an urban region. We will hang flyers for each phase of the study to advertise study details. A research assistant will pre-screen medical records of patients with clinic appointments and identify those who appear to be eligible. Written informed consent will be obtained from those interested in participating.

### Phase 1 procedures: qualitative methods to inform intervention adaptation

#### Overview

Individual interviews have been conducted with 29 treatment providers (n = 16 HIV providers; n = 13 substance use providers) and six PLWH who met eligibility criteria to gather feedback on the Life Steps protocol, ideas for adapting the protocol to PWUD, and training preferences. Interview topics included: (1) current support for HIV care within the clinic or community for HPWUD, (2) brainstorming intervention content, (3) critique of proposed intervention content and existing materials, and (4) evaluation of intervention length, intensity, frequency, and mode of delivery. Patient and provider data are being analyzed and triangulated to inform adaptation of the Life Steps intervention to HIV-infected PWUD. Patients and providers received $40 for participating in a 60–90 min qualitative interview.

#### Manual development

**Fig. 1:**
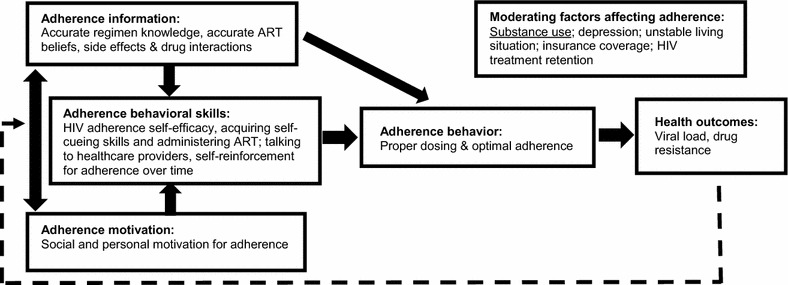
Information–motivation–behavioral skills model of ART adherence (Adapted from Fisher et al. [16])

The Life Steps manual will be enhanced to address the most salient needs of HIV-infected PWUD while retaining the core elements of the intervention. Data from Phase 1 will guide modification of the manual. The manual will be evaluated based on the theoretical model, targeted outcomes, therapeutic strategies, and content. *Substantive Changes Expected*. Based on extant literature, there are two key areas in which substantive changes are expected in the adaptation of the Life Steps manual (see Table 2): *Content*. The Information-Motivation Behavioral Model [16] will guide content targeting individual knowledge, motivation, and skills development (see Fig. 1). Considering the synergistic interaction of HIV and drug use on nonadherence, syndemics theory [1] will guide the adaptation of intervention content to address contextual challenges of HIV-infected PWUD (e.g., stigma, stress, poverty). *Delivery*. Based on the hypothesized outcomes from Phase 1, we expect the adapted LS-DU protocol will contain a new drug use module focused on building motivation for change and linkage to substance use treatment; this module will be informed by best practices in the BMI literature. While we have anticipated preliminary adaptations based on the extant literature, changes to the protocol will be based on data obtained during the qualitative phase which will be sufficiently broad and open to assess for factors that we have not anticipated. Once the manual is developed, a review panel of five experts in HIV, addiction, and ART adherence will provide feedback about the intervention manual. We will use an affinity grouping procedure to merge similar recommendations. The draft of recommendations will be reviewed through two rounds of preliminary ratings, teleconference discussions, and written comments to result in a set of final recommendations which will inform revision and finalization of the manual.

**Table 2:** Core elements of life steps and anticipated modifications

Module	Description	Anticipated modifications to life steps
*Existing life steps protocol*		
Step 1: Education and introduction	Information about how medication adherence plays in successful treatment is provided. The aim is to increase knowledge and self-efficacy to influence treatment success and introduce problem-solving for medication adherence	Information regarding the physiological and psychosocial impact drug use has in PLWH, address impact on ART medications and increased HIV transmission
Step 2: Transportation to appointments	Problem-solving strategies, brief cognitive restructuring, and rehearsal techniques are provided to address transportation issues to prevent missed appointments with health care providers	No anticipated modifications
Step 3: Obtaining medications	Develop a plan for continues access to medications. Address concerns about patient’s privacy and confidentiality during interactions with the pharmacist	No anticipated modifications
Step 4: Communicating with health care providers	Brief cognitive techniques are suggested for irrational fears about asking questions	Address concerns about drug use stigma; teach strategies for improving patient-provider relationship
Step 5: Coping with side effects	Learn to (a) pick a regimen with their doctor to minimize side effects; (b) re-interpret side effects as signs that medications are in their bloodstream and working; and (c) increase the salience of benefits of adherence	No anticipated modifications
Step 6: Formulating daily medication schedule	Patients complete a detailed map of an average day of pill-taking, specifying environmental and other cues for pill-taking throughout the day	Address challenges to maintaining the daily medication schedule during periods of intoxication
Step 7: Storing medications	Problem-solving techniques for storing medications when not at home	No anticipated modifications
Step 8: Cues for pill-taking	Taught how to use cues to remember to take pills (e.g., setting alarms, reminder system). Adaptive cognitions for adherence are rehearsed	No anticipated modifications
Step 9: Response to slips in adherence	Patients are taught how to handle slips and to avoid all-or-nothing thoughts. Cognitive techniques to cope with a lapse are discussed	Address how drug use may result in slips on adherence and how to minimize negative health outcomes
Step 10: Review	Review previous steps and address concerns	No anticipated modifications
*Anticipated additional adherence*-*related modules to the life steps protocol (based on the syndemics model by Singer* [1]*)*		
Interpersonal factors		
Increasing social support and relationship stability	Brief training in interpersonal skills and strategies to help increase support networks and build healthy relationships	
Individual factors		
Psychiatric comorbidities	Participants will receive a list of local mental health and substance abuse treatment options	
HIV-related beliefs	Address interaction toxicity beliefs in regards to taking ARTs while using drugs and alcohol	
Environmental factors		
Stigma	Cognitive techniques will be provided to help participants cope with stigma related to HIV and drug use	
Access to services	Participants will receive a list of local resources to assist with housing, transportation, and food insecurity	
*Anticipated additional drug use modules to the life steps protocol (based on the FRAMES approach by Miller and Sanchez *[23]*)*		
Personalized assessment and feedback	Participants will complete a baseline assessment of their drug use ad risk behaviors. Individualized feedback regarding personal risk or impairment will be given following assessment	
Changing risk behaviors	Use of MI techniques to address drug use and other risk behaviors. Techniques include assessing stage of change, use of decisional balance, address discrepancies between personal goals and behavior, and emphasize that autonomy and responsibility for change is explicitly on the participant	
Advice about changing	Advice about reducing or stopping drug use will be given to the participant in a nonjudgmental manner	
Treatment options	A menu of self-directed change options and treatment alternatives will be offered to the participant	
Empathic counseling	The clinician will show warmth, respect, and understanding towards the participant	
Self-efficacy for change	Self-efficacy to change drug use will be engendered in the participant to encourage change

### Phase 2 procedures: piloting and training of community-based facilitators

#### Therapist training, fidelity, and competence

Following manual refinement, two master’s-level community clinicians will be trained in fidelity and competence with the intervention protocol. Training will consist of a half-day didactic workshop, followed by video-taped role plays, which will be coded by two independent raters for fidelity and competence. We will assess competence using the Motivational Interviewing Treatment Integrity 2.0 (MITI) [17] and the Yale Adherence and Competency Scale [18]. Following training, clinicians will provide feedback regarding their training experience and recommendations for the training protocol.

#### Patient qualitative interviews

The two trained clinicians will deliver the LS-DU intervention to 8–12 HIV-infected PWUD using an iterative process. We will conduct exit interviews with patients and the two clinicians following completion of the intervention to assess intervention acceptability and feasibility. Data collected during the individual interviews will include: (1) critique of intervention content and materials, (2) evaluation of intervention length, intensity, frequency, and mode of delivery, and (3) suggestions to further enhance the intervention. Patients will again receive $40 for participating in a 60-min qualitative interview. Interviews will be administered until saturation is reached.

### Phase 3 procedures: type 1 hybrid effectiveness-implementation pilot trial

#### Overview

Following the open trial, a pilot type 1 hybrid randomized effectiveness-implementation trial will be conducted to determine the effectiveness of the LS-DU protocol relative to health education. Hybrid effectiveness-implementation trials are designs that simultaneously gather data on treatment effectiveness and implementation outcomes [19]. In a type 1 hybrid trial, the primary emphasis is on the evaluation of treatment effectiveness, and the secondary emphasis is on the collection of data on implementation outcomes. Because the current study is designed to collect pilot data for a future study, measures of acceptability and feasibility are defined as the primary outcomes. Measures of effectiveness are then defined as the secondary outcomes, while data on implementation effectiveness are gathered as an exploratory outcome.

All participants will complete a baseline assessment consisting of a series of self-report measures and a urine toxicology screen. Following completion of the baseline assessment, participants will begin 2 weeks of baseline ART adherence data collection using an electronic pill cap monitoring system (MEMScaps). Only after completing the MEMS data collection will patients be randomly assigned to treatment condition using urn randomization [20] controlling for gender and viral load.

#### Treatment conditions

Participants will be randomized to either the LS-DU experimental intervention or the health education control. We considered using the original Life Steps as a comparator condition; however, we believe that a minimally active, but ethical control group is the most direct test of the intervention (relative to treatment as usual). A more active control intervention, such as the original Life Steps protocol, may attenuate potential effects and would require a much larger sample size. The LS-DU intervention will utilize the manual refined at the end of Phase 2. Health education is a time-matched control condition that covers a variety of general health topics and has proven to be a credible control condition in prior studies [21]. Intervention clinicians will be given a health education training manual and will complete videotaped role plays. Two independent coders will assess fidelity to the health education intervention using a topic checklist. Both conditions will consist of two in-person intervention sessions of 60 min duration separated by 1 week. The LS-DU intervention will also consist of two follow-up booster phone sessions at 1 and 2 months following the last in-person session.

#### Assessments

With the exception of demographic data, all of the measures described below will be administered at baseline and the three follow-up assessments. To enhance retention, participants will receive an escalating compensation schedule in gift cards for completion of the 1-, 3-, and 6-month follow-ups. Participants will receive a breathalyzer test prior to each assessment, and impaired participants will be rescheduled.

The Information-Motivation Behavioral Model [16] has informed the battery of measures. The following basic demographic data will be collected: age, gender, race, ethnicity, current living situation, marital status, sexual orientation, educational attainment, employment status, date of HIV diagnosis and ART initiation, mode of transmission, substance use diagnosis, and the intensity and duration of the substance use disorder. Primary outcomes will be feasibility and acceptability of the LS-DU and assessment procedures to be used in a larger trial. We will assess preliminary effectiveness of the LS-DU in a real-world clinical setting delivered by trained community-based clinicians. Effectiveness outcomes will include ART adherence measured by MEMScaps, viral load, and substance use measured by the 30-day Timeline Followback. Implementation outcomes will include readiness to adopt and qualitative data on barriers and facilitators to uptake. We will also gather exploratory data on the following potential moderators of the LS-DU intervention: HIV treatment self-efficacy, depression, HIV treatment retention, and HIV and substance use problem behaviors measured by the Addiction Severity Index (see Table 3 for measures).

**Table 3:** Outcome variables and assessment points

Quantitative measures	Stakeholder	BL	1-, 3-mos follow-up	6-mos follow-up
Demographics	All	X		
*Acceptability*				
Program satisfaction questionnaire	Patients, clinicians			X
*Effectiveness*				
Medication adherence (MEMS)	Patients	X	X	X
Viral load	Patients	X		X
Substance use	Patients	X	X	X
*Implementation*				
Organizational readiness for change assessment tool	Clinic directors	X		
*Exploratory*				
HIV treatment self-efficacy	Patients	X	X	X
Depression	Patients	X	X	X
HIV treatment retention	Patients	X	X	X
Substance use risk behaviors	Patients	X	X	X

We will evaluate the implementation context and sustainability potential of the LS-DU at the clinic level through surveys and semi-structured interviews. Individual interviews will be conducted post-intervention with clinicians and clinic leadership. Data will be collected regarding perceptions of providers and leadership concerning barriers/facilitators to intervention adoption, tools needed to deliver the intervention consistently, resources needed to maintain the intervention long-term, and adaptations needed to integrate into regular practice. Providers and leadership will complete the Organizational Readiness for Implementing Change Scale [22] to examine organizational strengths/weaknesses that support sustainability of the intervention.

#### Quantitative data analysis plan

Considering this is a pilot project, analyses will have the goal of establishing feasibility and estimation of effect sizes, with modest expectations for rejection of the null hypotheses. As a first step, the equivalence of treatment condition assignment with regard to key baseline variables will be assessed on demographic characteristics, baseline ART adherence measures (self-report and viral load), drug category and addiction severity, and baseline levels of potential treatment mechanisms and moderators using tests of proportions or t-tests as appropriate. Should conditions differ on any characteristic; these variables will be used as covariates in outcome analyses. Other preliminary analyses will include patterns of missing data, research dropout rates, distributional properties of dependent and other measures, and correlations among outcome measures.

##### Hypotheses testing

Given the developmental nature of this study, our primary goal is to establish feasibility and acceptability of the LS-DU and assessment procedures to be implemented in a future larger scale hybrid type 2 randomized trial. Our primary hypothesis is that participants completing and community clinicians delivering the LS-DU intervention will report high levels of acceptability of the intervention. Our secondary hypothesis is that the LS-DU intervention will demonstrate greater effectiveness than the health education outcome in terms of promoting days of adherence to HIV medication and reducing substance use. We will also collect exploratory data on implementation potential of the intervention and moderators of implementation effectiveness. Due to the small sample size, we will primarily be hoping to find a pattern of results that is supportive of the LS-DU rather than rigorously testing hypotheses to determine a stable effect size.

##### Primary outcomes: acceptability

Participants’ mean ratings of the program satisfaction questionnaire will be examined to determine level of satisfaction of the LS-DU.

##### Feasibility

We will monitor the feasibility of the pilot trial by tracking recruitment, retention, and adherence rates of participants. We will assess feasibility of the intervention through qualitative interviews with the clinicians and a subset of patients.

##### Effectiveness

We will measure treatment effectiveness via adherence to HIV medication (MEMScaps), viral load (eligibility screener vs. baseline vs. 6-months), and days of drug use (Timeline Followback). Separate Generalized Estimating Equation (GEE) models for adherence will be generated for proportion of days on which study medication was taken (as indicated by MEMScap) and the proportion of expected pills taken at the 1-, 3-, and 6-month follow-ups. Examining interactions between intervention and time will test differences in adherence over the course of the intervention across the intervention conditions. As our sample size limits our ability to conduct sophisticated longitudinal analyses, we will use GEE with caution and will revert to more standard general linear models should we encounter model convergence problems. Additional measures of treatment effectiveness, including self-reported adherence, viral load, and drug use, will also be analyzed using GEE.

##### Implementation context and sustainability potential

Data will be collected at the clinic level through surveys and semi-structured interviews. Individual interviews will be conducted post-intervention with clinicians and clinic leadership. Data will be collected regarding perceptions of barriers and facilitators to intervention adoption, tools needed to deliver the intervention consistently, resources needed to maintain the intervention long-term, and adaptations needed to integrate into regular practice.

#### Qualitative analysis

Qualitative data will be analyzed using a thematic analysis framework and standard qualitative analysis techniques. An initial codebook will be developed from the interview guides and revised as themes emerge. Two coders will double-code all transcripts and discuss discrepancies until consensus is reached. A master codebook will be entered into NVivo 11. We will discuss the transcripts to analyze themes. A list of barriers and facilitators to intervention implementation and sustainability will be developed.

## Discussion

HIV-infected PWUD continue to experience poorer rates of ART adherence, lapses in treatment retention, and increased morbidity and mortality [7, 8]. Existing adherence interventions have yet to prove effective among this high-risk population. This protocol seeks to improve ART adherence and promote linkage to substance use treatment by developing an adherence intervention tailored to HIV-infected PWUD. This behavioral intervention will utilize the syndemics framework to address critical factors aimed at improving adherence and reducing substance use. This study will use a pilot type 1 hybrid effectiveness-implementation design [19] given that Life Steps and BMI have both proven efficacious. In order to speed the translation of research to practice, we test the effectiveness of these interventions delivered in a real-world clinical setting, while simultaneously examining potential barriers and facilitators to implementation.

While this study will advance our understanding of treatment science and clinical practice with HIV-infected PWUD, there are a number of limitations which should be noted. First, since this is a pilot study the sample size is small and, therefore, will require testing in a larger trial to further examine effectiveness should this study produce a pattern of results that is favorable to the LS-DU intervention. Second, this is a brief, first-line intervention that aims to improve ART adherence and link HIV-infected PWUD to substance use treatment. Consequently, this will not be a comprehensive treatment intervention, and patients might require more intensive substance use intervention post-linkage to care. Third, participants will not be blind to study purpose and intervention condition due to the informed consent process and the nature of the content being addressed in each behavioral condition.

This research will result in the development of an ART adherence intervention tailored for PWUD and combined with a BMI aiming to link patients to appropriate substance use treatment services. Data from this study regarding implementation and sustainability potential will result in the development of an implementation manual for community-based HIV clinical settings. This work will inform a larger, multi-site study that places equal emphasis on both effectiveness and implementation outcomes. These results have the potential to make a significant impact on the management of ART adherence by improving the quality and utilization of evidence-based adherence interventions delivered to HIV-infected PWUD in the clinic setting. From both a clinical and a public health perspective, there is an urgent need to develop effective interventions to improve ART adherence and reduce substance use among HIV-infected people who use drugs. To our knowledge, no research has yet to adapt a brief, theory driven protocol that integrates HIV and substance use treatment among this high-risk population.

## Abbreviations

HIV: human immunodeficiency virus
ART: antiretroviral therapy
PWUD: people who use drugs
PLWH: people living with HIV
CBT: cognitive behavior therapy
BMI: brief motivational interview
LS-DU: Life Steps-Drug Use
